# Depression, Anxiety and Mental State in Patients with Early Stage Dementia: Comparison of Reminiscence Therapy and Cognitive Stimulation Therapy

**DOI:** 10.1002/alz.095482

**Published:** 2025-01-09

**Authors:** Zeynep Gümüş Demir, İdil Arasan Doğan, Remziye Keskin, Defne Demet Koyutürk

**Affiliations:** ^1^ Uskudar University, Istanbul, Uskudar Turkey; ^2^ Darulaceze Presidency, İstanbul, Uskudar Turkey

## Abstract

**Background:**

It was aimed to compare Reminiscence and Cognitive Stimulation Therapies in terms of depression, anxiety symptoms and mental status in elderly individuals with early stage dementia in institutional care.

**Method:**

**T**he design of the study was an experimental model with pretest‐ posttest control group. A total of 24 elderly individuals, 13 men and 11 women, (n = 12) for the study group and (n = 12) for the comparison group, constituted the sample of the study. All of the sample were elderly individuals with early stage dementia from long‐term care facility residents in Istanbul. Sociodemographic Information Form, Geriatric Depression Scale, Beck Anxiety Scale and Standardized Mini Mental Test were applied to the participants. The study group received 10 sessions of Reminiscence Therapy once a week, each session lasting 90 minutes, while the comparison group received Cognitive Stimulation Therapy under the same conditions.

**Result:**

As of the findings of the study, it was observed that depression and anxiety levels in the Reminiscence Therapy group showed a significant regression compared to the pre‐test findings, while the depression level was found to be higher than the Cognitive Stimulation Therapy group at the beginning of the application. A decrease in depression levels was also found in the Cognitive Stimulation Therapy group. The anxiety level of the group, which showed a downward trend compared to the pre‐test, was found to be more resistant than the anxiety level of the Reminiscence Group in this context. When the Standardized Mini Mental Test findings were evaluated, it was observed that the cognitive function levels increased in the group receiving Cognitive Stimulation Therapy, while no significant change was found in the Reminiscence Therapy Group.

**Conclusion:**

This result reveals the critical role of Cognitive Stimulation Therapy in terms of cognitive functions. Although quantitative studies on individuals with dementia are quite rare, this study is important in terms of the fact that elderly individuals with dementia in institutional care carry out a participatory process with other elderly individuals with dementia, the effect of psychotherapy models applied in this context on psychological symptoms is seen and thus sets an example for positive interventions that can be made.

